# Identification of Priority Conservation Areas and Potential Corridors for Jaguars in the Caatinga Biome, Brazil

**DOI:** 10.1371/journal.pone.0092950

**Published:** 2014-04-07

**Authors:** Ronaldo Gonçalves Morato, Katia Maria Paschoaletto Micchi de Barros Ferraz, Rogério Cunha de Paula, Cláudia Bueno de Campos

**Affiliations:** 1 Centro Nacional de Pesquisa e Conservação de Mamíferos Carnívoros - Instituto Chico Mendes de Conservação da Biodiversidade - Ministério do Meio Ambiente, Atibaia, São Paulo, Brazil; 2 Instituto para a Conservação dos Carnívoros Neotropicais, Atibaia, São Paulo, Brazil; 3 Departamento de Ciências Florestais, Escola Superior de Agricultura “Luiz de Queiroz”, Universidade de São Paulo, Piracicaba, São Paulo, Brazil; Federal University of Parana (UFPR) ) – Campus Palotina, Brazil

## Abstract

The jaguar, *Panthera onca*, is a top predator with the extant population found within the Brazilian Caatinga biome now known to be on the brink of extinction. Designing new conservation units and potential corridors are therefore crucial for the long-term survival of the species within the Caatinga biome. Thus, our aims were: 1) to recognize suitable areas for jaguar occurrence, 2) to delineate areas for jaguar conservation (PJCUs), 3) to design corridors among priority areas, and 4) to prioritize PJCUs. A total of 62 points records of jaguar occurrence and 10 potential predictors were analyzed in a GIS environment. A predictive distributional map was obtained using Species Distribution Modeling (SDM) as performed by the Maximum Entropy (Maxent) algorithm. Areas equal to or higher than the median suitability value of 0.595 were selected as of high suitability for jaguar occurrence and named as Priority Jaguar Conservation Units (PJCU). Ten PJCUs with sizes varying from 23.6 km^2^ to 4,311.0 km^2^ were identified. Afterwards, we combined the response curve, as generated by SDM, and expert opinions to create a permeability matrix and to identify least cost corridors and buffer zones between each PJCU pair. Connectivity corridors and buffer zone for jaguar movement included an area of 8.884,26 km^2^ and the total corridor length is about 160.94 km. Prioritizing criteria indicated the PJCU representing c.a. 68.61% of the total PJCU area (PJCU # 1) as of high priority for conservation and connectivity with others PJCUs (PJCUs # 4, 5 and 7) desirable for the long term survival of the species. In conclusion, by using the jaguar as a focal species and combining SDM and expert opinion we were able to create a valid framework for practical conservation actions at the Caatinga biome. The same approach could be used for the conservation of other carnivores.

## Introduction

Habitat fragmentation has been recognized as a major threat to the conservation of a variety of species [1] [2] mainly because it can isolate previously connected populations and, consequently, disrupt original patterns of gene flow likely to lead to drift-induced differentiation among local population units [3]. For this reason, corridors are considered a valuable conservation tool [4] to promote the ability of individuals to move among habitat patches [5] and provide, in this way, an opportunity to mitigate the negative effects of demographic and environmental stochasticity [6] [7] and to sustain the population's genetic diversity and maintain the evolutionary processes associated [8].

Connectivity is a key factor supporting the long-term survival of a variety of species in fragmented areas. However, designing corridors has been a challenge due to the lack of methodological examples found in the literature, no widely accepted protocols, and few available practical examples of field assessment of wildlife corridors [9].

Different approaches have been used for designing corridors, with most of them based on target species and taking into account the behavioural response of these organisms to the landscape structure. Patterns of animal movement may be used as the baseline for corridor design; however, it depends on time-consuming methods, such as the use long-term field data, dispersal movements, and demographics [10]. In this way, using models that rely solely on presence data to evaluate a species potential distribution and identify high suitable areas for a focal species could be a very useful tool for building “potential corridors” [11] [12]. In general this information can be applied for identifying core populations or habitat [11], which could be connected. In addition, these models could estimate the probability of a species occurrence related to different environmental variables [12]. Considering that some population models frequently used to evaluate connectivity, such as the least-cost path analyses models, depend on an understanding of how animals move through a landscape [13] such information can indicate environmental factors facilitating or impeaching animal movement or survival.

Large carnivores are often proposed as focal species when evaluating landscape connectivity [10] due to their large area requirements [14] and because their dispersal through a landscape is frequently limited or blocked by areas of high human development or access [15].

The jaguar (*Panthera onca*), the largest cat of the Americas, has a broad distribution throughout Central and South America [16]. It is considered a focal species since its survival requirements encompass multiple factors that are essential for maintaining an ecologically healthy environment [17]. Recent research indicates that the reduction of a focal species population size, such as the jaguar, can lead to the extinction of another species in the community [18]. In this way, a range-wide model of landscape connectivity has been proposed using the jaguar as a focal species [19]. Besides the importance of this framework, we state the need of continuing studies at regional or local level. Also, it is important to mention that jaguars can occupy different habitat types and the use and selection of this space can be influenced by a variety of factors across its distribution range. In this way, connectivity models, using the jaguar as a focal species, should consider factors affecting its behaviour at more refined scales.

We focused this study in the Caatinga biome, considered a priority area for jaguar conservation since its population is listed as critically endangered [20]. Considering the entire jaguar distribution the Caatinga biome represents one of the few Xeric type regions where jaguars still persist. In addition, this kind of habitat is atypical for the jaguar where the species remains poorly studied [21]. The Caatinga biome encompass an area of 844,453 km^2^ and represents 9.9% of the Brazilian territory [22], however only 7.3% of this biome falls within the boundaries of protected areas and only 1% is within any strictly protected Conservation Unit [23], making urgent the establishment of strategies for biodiversity conservation in this region. Until recently, jaguar occurrence was supposed to be restricted to 0.1% of the Caatinga biome, within the Serra da Capivara National Park (1,000 km^2^) representing the unique jaguar core population in the biome which probability of long-term survival was considered low [24]. However, recently we reported jaguar presence [25] on areas where it had been thought to be long extirpated. By taking the jaguar as our focal species in the Caatinga biome, the objectives of this study were: 1) to recognize suitable areas for jaguar occurrence; 2) to delineate areas for jaguar conservation (hereafter PJCUs); 3) to design corridors among priority areas; 4) to prioritize PJCUs. Although the expected results focus on jaguar in the Caatinga biome, the methodology and conclusions drawn present a model for conservation planning that could be applied to other areas of jaguar distribution and also to other widely ranging species.

## Methods

### Study area

**Figure 1:**
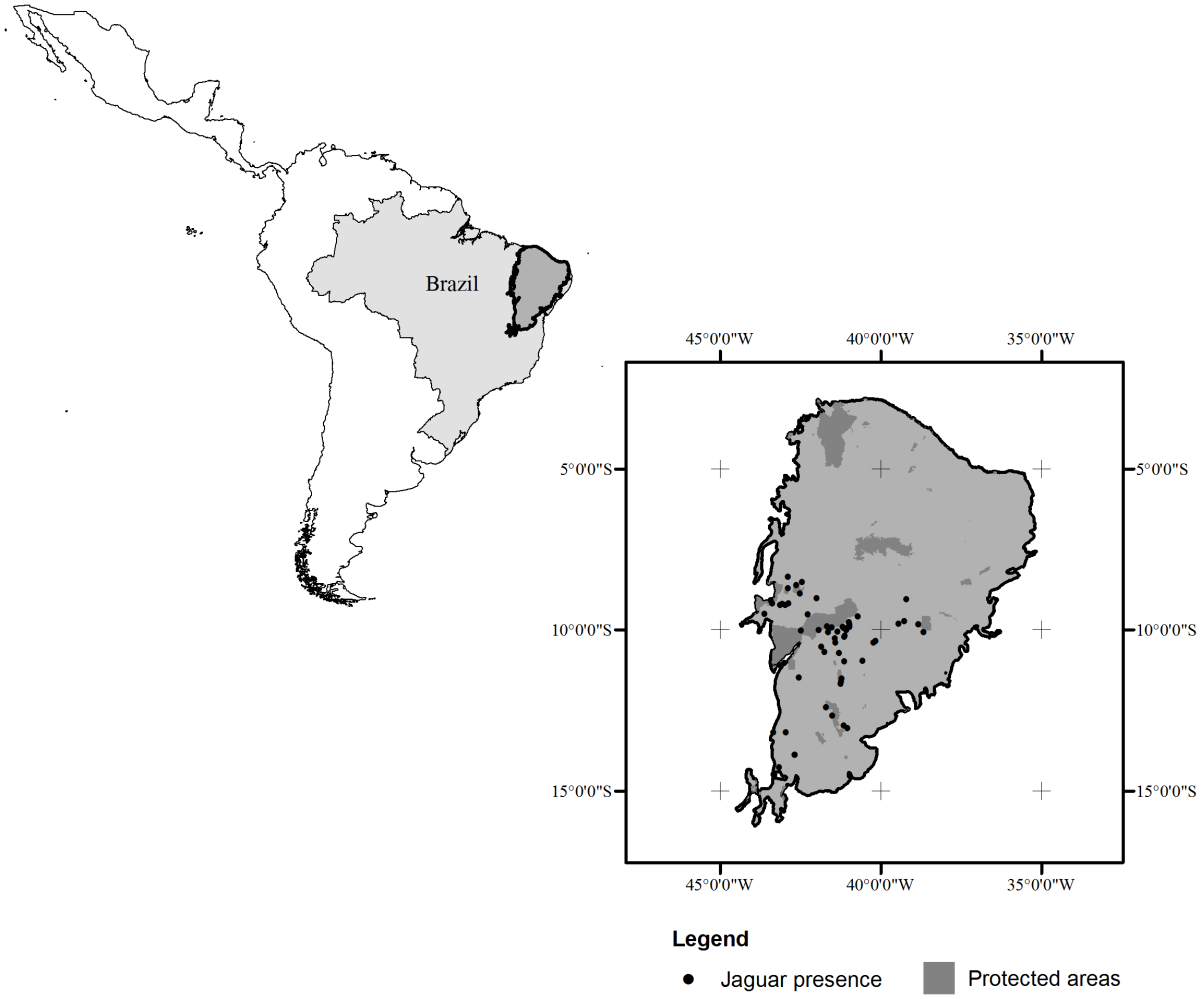
Location of Caatinga biome in Brazil, protected areas in the Caatinga biome and the presence data used for modeling.

This study was carried out in the Caatinga biome (844,453 km^2^), arid and semi-arid regions extending across eight states of Brazil: Bahia, Sergipe, Alagoas, Pernambuco, Paraíba, Rio Grande do Norte, Ceará, Piauí, and extreme north of Minas Gerais [26] (Figure 1). Xerophytic vegetation type dominated the Caatinga, characterized by spiny deciduous shrubs and trees in association with succulent plants, cacti and bromeliads [27]. In agreement with Andrade-Lima [28], there are twelve Caatinga types distributed in seven physiognomies and six physical units. Annual rainfall may vary from close to zero to as much as ten times the long-term annual average and deviation from the normal rainfall may be higher than 55%. Usually, 20% of the annual rainfall occurs on a single day and 60% in a single month [28] [29]. Most rain falls between September and March. Average annual rainfall is 644 mm, with a 50-year maximum of 1,131 mm and minimum of 250 mm [30]. Mean annual temperature is 27.6°C.

### Species Distribution Modeling

The Species Distribution Modeling (hereafter SDM) for jaguar occurrence in Caatinga biome was generated by the maximum entropy algorithm, as implemented in Maxent software 3.3.3e [31] [32]. Maxent is a recently introduced modeling technique, achieving high predictive accuracy and enjoying several additional attractive properties [32]. The idea of Maxent is to estimate a target probability distribution by finding the probability distribution of maximum entropy (i.e., that is most spread out, or closest to uniform), subject to a set of constraints that represent our incomplete information about the target distribution. When Maxent is applied to presence-only species distribution modeling, the pixels of the study area make up the space on which the Maxent probability distribution is defined [31]. Different studies have demonstrated the utility of species distribution modeling to identify areas of high conservation value, as performed by Maxent [12] or ensemble models [11], with Maxent showing, in general, best performance [11] [33] [34] [35] [36] [37].

Models were generated using presence-only data (N = 62) (Table S1; Figure 1) and environmental variables (Table 1) at a spatial resolution of 0.0083 decimal degree (∼1 km^2^). We selected functionally relevant variables for the species [38], avoiding the autocorrelation. We considered climatic and topographic factors assumed to be important to determine the jaguar distribution, as previously reported [11] [40]. We add two factors that have been reported to be important to determine jaguar presence in the Caatinga biome: distance from water [41] and precipitation of driest month as reported by local people. All presence records were obtained from National Predator Center (CENAP-ICMBio) database and literature [42] [43]. All runs were set with a convergence threshold of 1.0^E–5^ with 500 iteractions and with 10,000 background points, auto features, and analysis of variable importance measured by Jackknife, response curves and random seed.

**Table 1:** Environmental variables used for Species Distribution Modeling (SDM) for jaguar at Caatinga biome, Brazil.

Variables	Dataset name	Spatial Resolution	Year	Source
Land cover	GlobCover Land Cover version v2.3	300 meters	2009	ESA GlobCover 2009 Project
Elevation	Global elevation data	30 arc-second	2004	NASA Shuttle Radar Topography Mission
Distance from water	Gradient distance from vetor map from water	1∶5,000,000	2004	Brazilian Institute of Geography and Statistics (IBGE)
Bioclimatic variables	Bio1 = Annual mean temperature	30 arc second	2005	Data layers from Worldclim global climate variables
	Bio2 = Mean diurnal range*			
	Bio5 = Max temperature of warmest month			
	Bio6 = Min temperature of coldest month			
	Bio12 = Annual precipitation			
	Bio13 = Precipitation of wettest month			
	Bio14 = Precipitation of driest month			

*mean of monthly (max temp - min temp).

The SDM was generated by bootstrapping methods with 10 random partitions with replacements using 70% of the dataset for training and 30% for testing models [44]. The average model was cut off by the 10 percentile training presence logistic threshold (0.2613) as it provided the best accurate model for the species occurrence in the biome. We tested the SDM's predictive ability for jaguar occurrence in the Caatinga biome by plotting a new independent dataset not used for modeling (N = 38; Table S2) from recent species occurrence points.

The SDM was evaluated by AUC value, binomial probability and omission error [44] [45].

### High Priority Areas for Conservation

We used a different approach from that proposed by Sanderson et al. [24] to identify jaguar conservation units. From the SDM, we selected areas equal to or higher than the median suitability value of 0.595, which represents areas of high suitability for jaguar occurrence [11]. Then, we used the percent volume contour (i.e., raster layer representing a probability density distribution) from Kernel tools in Hawth's analysis tools for ArcGis [46] to delimit these areas, which we named as Priority Jaguar Conservation Units (PJCUs) (i.e., continuous areas of high suitability for jaguar occurrence).

### Corridors Modeling

**Figure 2:**
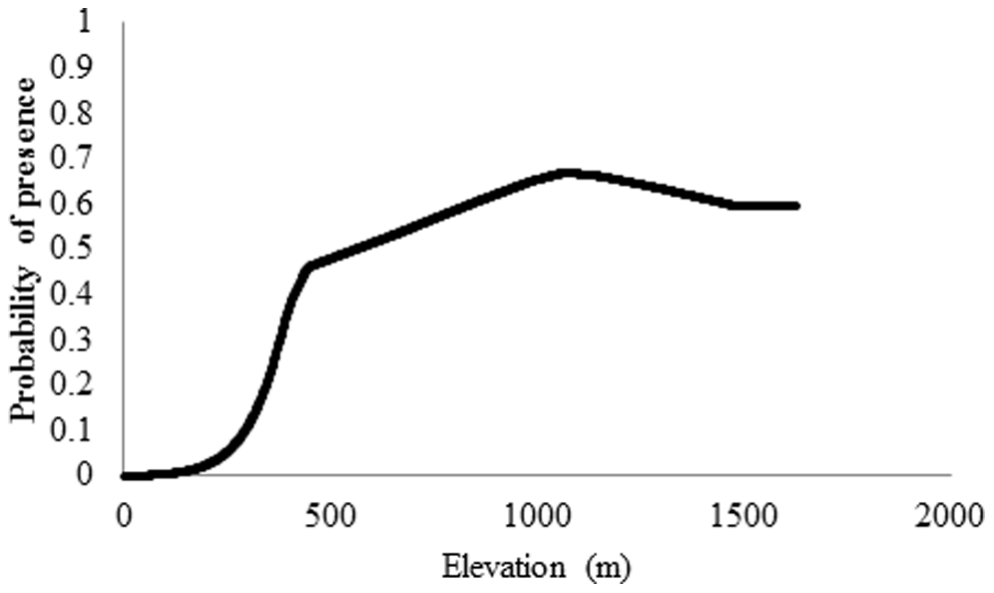
Marginal response curve of altitude, the variable that contributed most to the SDM of jaguar occurrence at the Caatinga biome.

Connectivity modeling was performed among PJCUs as proposed by Rabinowitz and Zeller [19]. We defined five predictors (Table 2) for creating the cost surface or permeability matrix (Table 3) and attributed cost values (ranging from 0 – no cost for jaguar movement – to 10 – high cost for jaguar movement) for each according to Rabinowitz and Zeller [19]. Cost values for elevation, the variable that contributed substantially to the SDM, were attributed based on the marginal response curve provided by the SDM (Figure 2). Following the procedures proposed by Rabinowitz and Zeller [19], we used the Cost-Distance function (Spatial Analyst, ArcGis 9.3) to delineate movement cost grids for each PJCU. After, we used the cost-distance grids as inputs for the Corridor function in Spatial Analyst for all proximate pairs of PJCUs, resulting in least-cost corridors among each pair. Then, we used the minimum mosaic method, combining all overlapping corridors to generate the final least-cost corridor model. Finally, differently from Rabinowitz and Zeller [19], we used the cost path function with cost-distance grids and PJCUs as inputs to calculate the least-cost path from a source to a destination. Crossing the least-cost paths to least-cost corridor model we then selected the best routes, hereafter named corridors, for jaguar dispersal through surfaces with no or low cost for movement. In addition, we identified “buffer zones” around PJCUs and corridors.

**Table 2:** Geographical databases used for connectivity modeling.

Variable	Dataset name	Spatial resolution or scale	Year of data	Source
Land cover	GlobCover Land Cover version v2.3	300 meters	2009	ESA GlobCover 2009 Project
Elevation	Global elevation data	30 arc-second	2004	NASA Shuttle Radar Topography Mission
Human Population density	Gridded population of the world v3	2.5 min	2010	Center for International Earth Science Information Network (CIESIN)
Distance from settlements	Gradient distance from vetor map from settlements	1∶5,000,000 scale	2004	Brazilian Institute of Geography and Statistics (IBGE)
Roads	Gradient distance from vector map from roads	1∶5,000,000 scale	2004	Brazilian Institute of Geography and Statistics (IBGE)

**Table 3:** Classes of landscape layers and cost values for jaguar movement.

Landscape cover			Elevation (m)		Human Population Density (inhabitants/km^2^)		Distance from roads (km)		Distance from settlements (km)	
ID	Classes	Cost values	Classes	Cost values	Classes	Cost values	Classes	Cost values	Classes	Cost values
14	Rainfed croplands	2	0–250	7	0–20	1	0–2	7	0–2	8
20	Mosaic cropland (50–70%)/vegetation (grassland/shrubland/forest) (20–50%)	4	250–500	6	20–40	5	2–4	4	2–4	5
30	Mosaic vegetation (grassland/shrubland/forest) (50–70%)/cropland (20–50%)	6	500–750	4	40–80	7	4–8	2	4–8	4
40	Closed to open (>15%) broadleaved evergreen or semi-deciduous forest (>5 m)	2	750–1000	2	80–160	9	8–16	1	8–16	1
50	Closed (>40%) broadleaved deciduous forest (>5 m)	2	1000–1700	0	160–320	10	>16	0	>16	0
60	Open (15–40%) broadleaved deciduous forest/woodland (>5 m)	0			>320	BA				
110	Mosaic forest or shrubland (50–70%)/grassland (20–50%)	4								
120	Mosaic grassland (50–70%)/forest or shrubland (20–50%)	4								
130	Closed to open (>15%) (broadleaved or needleleaved, evergreen or deciduous) shrubland (<5 m)	0								
140	Closed to open (>15%) herbaceous vegetation (grassland, savannas or lichens/mosses)	4								
150	Sparse (<15%) vegetation	4								
160	Closed to open (>15%) broadleaved forest regularly flooded (semi-permanently or temporarily) - Fresh or brackish water	4								
170	Closed (>40%) broadleaved forest or shrubland permanently flooded - Saline or brackish water	4								
180	Closed to open (>15%) grassland or woody vegetation on regularly flooded or waterlogged soil - Fresh, brackish or saline water	4								
190	Artificial surfaces and associated areas (Urban areas >50%)	4								
200	Bare areas	4								
210	Water bodies	4								

Costs values ranged from 0 (no cost for jaguar movement) to 10 (high cost for jaguar movement). BA means barrier for jaguar movement.

### PJCUs categorization

For categorizing PJCUs we considered the follow aspects, in order of importance: 1) PJCU size; 2) connectivity, and; 3) jaguar population status [24]. For PJCU size we estimate the smallest continuous area necessary to preserve a viable population of 50 individuals [24] as suggested by Rodriguez-Soto et al. [11]. In brief, we assumed (1) a sex ratio of at least one male every two females [47] [48] and thus counting on 15 males and 35 females, (2) an average home range of 130 km^2^ for males and 41 km^2^ for females [41] and (3) a complete overlap of the home range of one male with two females [49]. In this way the smallest continuous area necessary to preserve a viable jaguar population corresponds roughly to 1,700 km^2^ of high suitability habitats. In this way, PJCUs≥1,700 km^2^ received three points. Areas smaller than 1,700 km^2^ but with adequate habitat where jaguar populations can increase if threats were alleviated received two points. Finally, areas that cannot hold a jaguar population but still can function as stepping stone areas received one point. For connectivity, each PJCU received one point for each possible connection. Considering the jaguar population status, we combined the PJCU size previously calculated, with density estimate (1.57±0.43) previously reported by Sollmann et al. [21] (Table 4). Despite other available densities, Sollmann et al. [21] presented a spatially explicit capture-recapture model resulting in more precise estimates [50] than previously published non-spatial estimates [51] [52]. PJCUs containing at least 50 individuals, considering it to be genetically stable for 100 years [24], received three points, PJCUs containing fewer than 50 individuals but still can increase if threats can be reduced [24] received two points. PJCUs where the smaller estimated population is less than 1.0 but still can function as stepping stone areas received one point. Arbitrarily, we defined PJCUs with 8–9 points as high priority, PJCUs between 5–7 points as medium priority and PJCUs with 3–4 points as low priority.

**Table 4:** Priority Jaguar Conservation Units (PJCUs) identified in the Caatinga Biome.

PJCUs	Area (km^2^)	Mean estimated population size (minimum-maximum)	Number of possible connections	Priority values (points)	Priority Status
1	4311.0	67.7 (49.1–86.2)	3	9	High
2	1053.7	16.5 (12.0–21.0)	1	5	Medium
3	386.3	6.1 (4.4–7.7)	1	5	Medium
4	264.0	4.1 (3.0–5.2)	3	7	Medium
5	82.7	NA	2	4	Low
6	46.5	NA	2	4	Low
7	45.5	NA	2	4	Low
8	29.4	NA	1	3	Low
9	40.5	NA	1	3	Low
10	23.6	NA	1	3	Low
**Total**	**6,283.2**	**94.4 (68.52–120.1)**			

Total area, estimated population size and connectivity were used to prioritize the PJCUs.

NA = smaller estimated population is less than 1.0.

## Results

**Figure 3:**
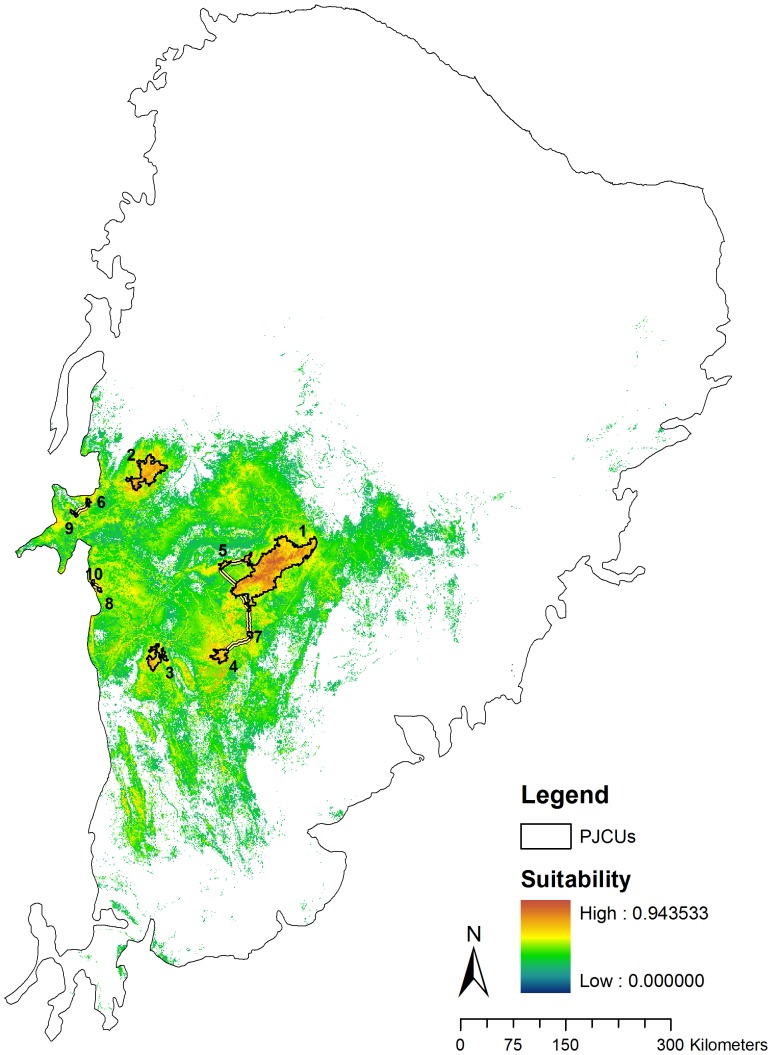
Jaguar distribution model and the Priority Jaguar Conservation Units (PJCUs) with high suitability areas (equal to or higher than the median suitability value of 0.595) (in detail).

The SDM for jaguar at Caatinga biome (Figure 3) was highly significant (AUC = 0.882±0.028, omission error = 0.283, p<0.001). The model also was highly accurate: 97% of the new independent data set was correctly predicted by the model and 52.94% of the presence points were predicted in highly suitable areas (≥70%). Elevation (27.34%) was the variable that most influenced jaguar presence in the Caatinga biome (Figure 2). The suitable area for jaguar occurrence in the Caatinga biome encompasses a total of 155,544 km^2^ (18.46% of the total biome). This area is composed mostly by closed to open shrubland (50.87%; 79,130 km^2^).

We identified ten PJCUs (6,283.2 km^2^) that represented areas of high environmental suitability for jaguar occurrence at the Caatinga (Figure 3). PJCU #1 represented approximately 68.61% of the total PJCUs area and could sustain a population of 67.7 (49.1–86.2) individuals (Table 4). Five PJCUs (#1, 3, 5, 8, 10) predominantly encompassed the closed to open shrubland, which is the main land cover type in both the Caatinga biome (31.81%) and the potential distribution area for jaguar occurrence (50.87%).

**Figure 4:**
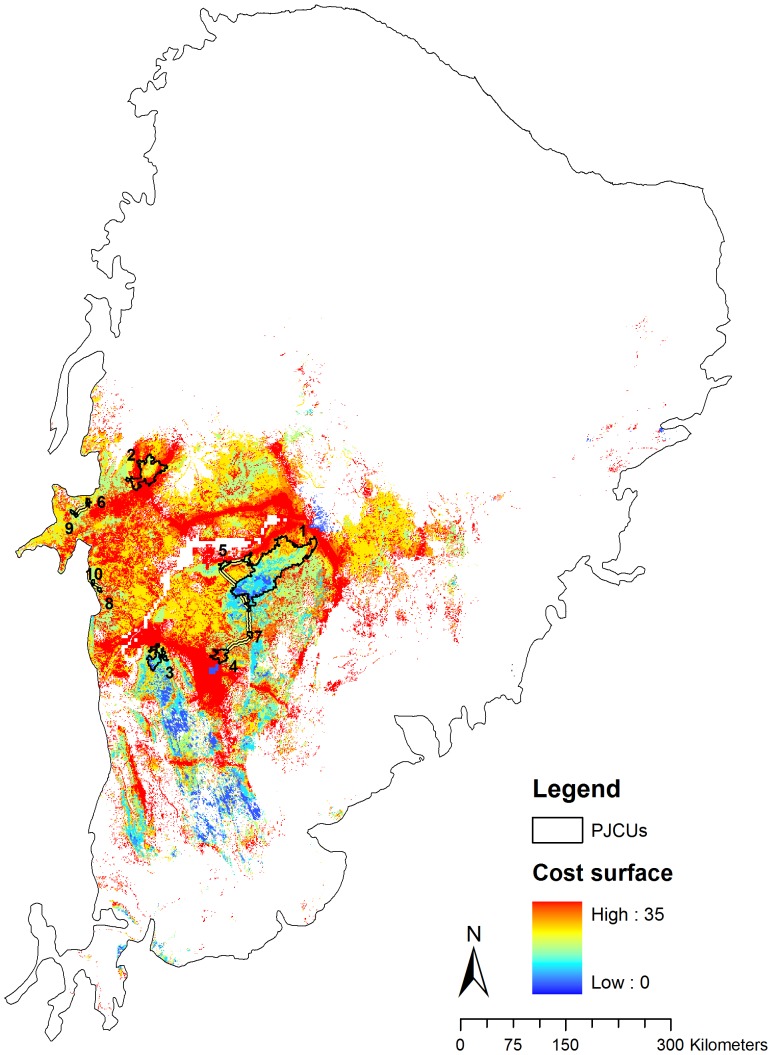
Cost surface for jaguar movement in the Caatinga biome with the Priority Jaguar Conservation Units (PCJUs). The higher the value of the cost surface, the less permeable is the pixel for jaguar movement.

**Figure 5:**
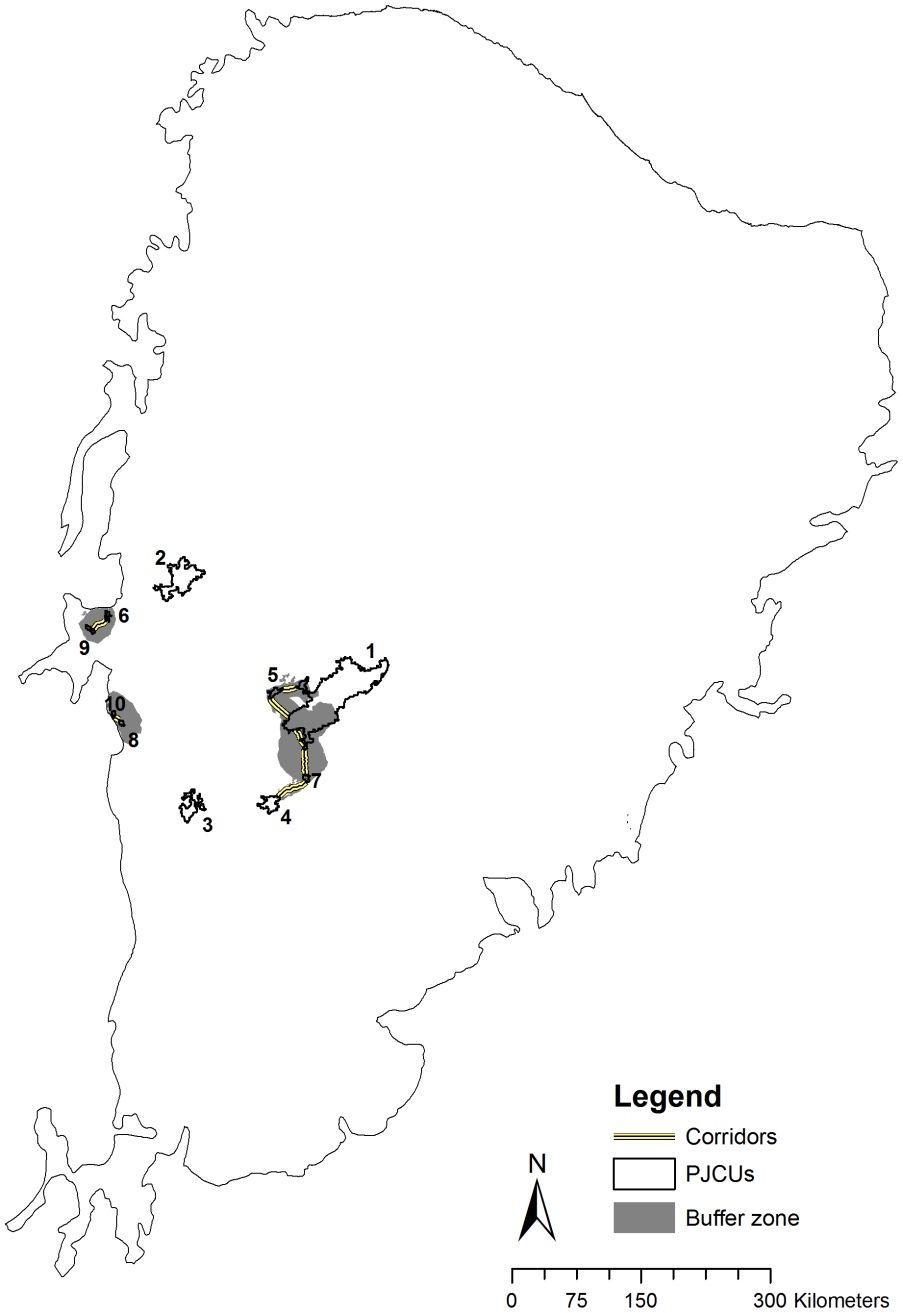
Connectivity corridors and buffer zones for jaguar movement and dispersal among the Priority Jaguar Conservation Units (PJCUs) in the Caatinga biome.

Connectivity modeling revealed high permeability or low cost surface around most PJCUs (Figure 4 and 5). The least-cost corridor analysis indicated three groups of well-connected PJCUs. The first and the biggest group (PJCUs #1, 5, 4 and 7) contained approximately 74.80% of the total area of all PJCUs. The second (PJCUs #9 and 6) and third (PJCUs #8 and 10) groups contained about 19% of the total area. All the three groups are isolated from each other. Modeling also revealed two PJCUs (#2 and 3) with no connections to any other PJCU.

Connectivity corridors and buffer zone for jaguar movement (Figure 5) included an area of 8,884.26 km^2^, encompassing 50.89% (∼4,524.3 km^2^) of closed to open shrubland. The area also included 13.22% (∼1,175 km^2^) of a mosaic with predominance of cropland, and less than 50% of grassland, shrubland or forest, and 11.61% (∼1,032.5 km^2^) of an open (15–40%) broadleaved deciduous forest. The corridors for jaguar dispersal (Figure 5) totalize about 160.94 km.

## Discussion

We identified high priority or core areas for jaguar conservation in the Caatinga biome by using the SDM. In addition we were able to identify feasible corridors by connectivity modelling. Our model increased the total suitable area for jaguar to almost seven times than previously reported by Sanderson et al. [24]; similar results were reported in Mexico after applying species distribution model techniques [11]. In addition to a core area previously described by Sanderson et al. [24] and Zeller [53], our model identified nine new highly suitable areas where the size varies from 23.6 km^2^ to 4,311.0 km^2^. Different from those authors, we used SDM to identify “core areas” with 62 point locations distributed in the biome, compared with five restricted to Serra da Capivara National Park previously described by Sanderson et al. [24]. Since this first report, further scientific studies in the field [25] [42] [43] and literature reviews [54] [55] have been performed, resulting in a higher number of jaguar point locations and better knowledge of the Caatinga's fauna [56].

Except for PJCUs # 8 and 10, jaguars have been reported in or near all the PJCUs. It is clear that most PJCUs cannot sustain a long-term viable population (see Table 4), considering 50 individuals living in a suitable habitat [24]. However, for conservation purposes, we also need to consider the potential connectivity between the PJCUs to manage it as a unique population. In this way, even small patches can function as stepping stone islands, where jaguars can feed or rest, facilitating the migration of dispersal individuals [57] that, sometimes, can travel over 1,607 km [19]. In addition, we need to reinforce the fact that the Caatinga biome has only 1% of strictly protected areas [23] and any additional unit can be important for the conservation of other species.

Despite the suitability of the 18.46% biome to jaguar occupancy, less than 1% is considered of high probability of occurrence (the PJCUs) as indicated by our model. We consider that the status of jaguar populations and their occupancy in the biome reflects the situation of the environment itself. The Caatinga is under severe threats due to an unsustainable land use such as unplanned expansion of croplands and cattle ranching activities, mining and eolic energy matrix [58] [59]. Jaguar is a sensitive species to human activities being subject to an inappropriate land use [39].

Jaguars in the Caatinga biome seem to be isolated from other populations. There is no recent report of jaguar presence in the northern part of the Caatinga suggesting that contact with the Amazon population is disrupted. Connectivity with the Atlantic Rain Forest seems to be unfeasible at this moment, since important anthropogenic factors, such as human density, can impeach jaguar movement in these areas. In fact, Rabinowitz and Zeller [19] described these areas as corridors of concern indicating that more investigation is required to verify jaguar movement between the Caatinga and the Atlantic Rain Forest. Moreover our recent survey in the east part of the Caatinga did not report jaguar presence (data not shown), which corroborates the indication of an ongoing local extinction in the last 10 years [60]. The only possible connection of Caatinga's jaguar populations would be with the Cerrado biome through the western PJCU's (# 6, 8, 9 and 10). The PJCU group composed by # 8 and 10 is somewhat far from viable jaguar populations from the Cerrado due to the expansion of crop fields in the savannas [61]. Feasible possibilities of connections with the Cerrado's populations are limited to the PJCU group composed by # 6 and 9 that might contact other populations due to a large mosaic of remaining natural areas. In other hand, this group is still isolated from the others Caatinga's PJCUs. Nevertheless, further investigation on the western area is necessary to verify the status and movements of jaguars in this region. Furthermore, we expected that the PJCU # 2 would play an essential role in the Caatinga's jaguar conservation, as previously reported by Sanderson et al. [24]. However, our model indicated that this PJCU is completely isolated corroborating a recent study that showed signs of reduced gene flow between jaguars from Serra da Capivara National Park (PJCU #2) and other regions [62].

Considering the jaguar critical status in the Caatinga biome [20] the population isolation can perform a final stage to the species extinction in the biome. In this way, the implementation of our corridor proposal represents a crucial alternative to long-term preservation of the Caatinga's jaguar population. However, strategies to ameliorate the negative effects of this isolation, such as habitat restoration [63] population supplementation and reintroductions [64] should be considered.

For our purposes, Maxent has the advantage of generating response curves of the predicted probability of occurrence for the jaguar facing different variables, where final results were used to construct the permeability matrix for connectivity modelling. In this way, our elevation cost values differed from those reported by Rabinowitz and Zeller [19]. In this study, higher elevation (1000 to 1700 m) is favoring jaguar presence in the Caatinga biome (see Figure 2). On the contrary, the jaguar detection probability is higher in lower elevation areas of the Nicaragua forests [9]. Two factors can explain our findings: 1) high elevation areas have low human density and also very restricted access to people, as consequence low human activity. Besides we did not use the human density and activities as layers in our model, overlapping human settlements maps from Instituto Brasileiro de Geografia e Estatística [61] with our final model corroborate our hypothesis. Jaguars, in general, avoid disturbed areas [39] [65] [66] [67] [68] and anthropogenic land uses can negatively affect jaguar presence [69]; 2) most of the high elevation areas are covered by the main vegetation types favoring jaguar presence. Precipitation in the driest month seems to play an important role for jaguar presence in this arid and semi-arid region. During the dry season natural holes can store water for large periods, however not for the entire season. In this way, we can speculate that occasional rains will “refill” this water sources avoiding animals moving long distances searching for it. It is in accord with Astete [41] findings since the author reports the positive influence of waterholes in the jaguar presence at Serra da Capivara National Park.

Our final model is primarily based on a focal species, presence-only data and posteriori least-cost patch analysis. The construction of the permeability matrix followed the model proposed by Rabinowitz and Zeller [19] with two differences: 1) elevation classes and values were built based on the response curves of the predicted probability of jaguar occurrence, and; 2) land cover values were based on experts' opinions working in the biome, resulting in different values used in Rabinowitz and Zeller [19] model. Closed to open broadleaved evergreen or semi-deciduous forest and open (15–40%) broadleaved deciduous forests were the main land cover types facilitating jaguar movement and/or dispersal, according to expert opinions. It differs from Rabinowitz and Zeller [19] and Rodriguez-Soto et al. [11] that reported lower probability of jaguar occurrence in these types of land cover.

Costs for creating national parks or any other type of protected area can be extremely high and prioritizing this action can help decision makers. Based on the prioritization criteria we applied, the PJCU # 1 has high priority while PJCUs #2, 3, 4, are of medium priority and PJCUs # 5, 6, 7, 8, 9 and 10 of low priority for jaguar conservation in the biome. Unfortunately, PJCU #1 area is not strictly protected and also is not included in any protected area category according to the Brazilian protected areas system [70], instead this area has been claimed as a potential area for installing an Eolic energy matrix and mine exploitation [59]. PJCUs # 2 (Serra da Capivara National Park), 6 and 9 (Serra das Confusões National Park) are strictly protected by law. A potential corridor between the PJCUs # 2 and 6 (not identified by the model) has already been implemented by the Brazilian government. The lack of connectivity between the PJCU # 2 and the rest is of major concern since this has been considered as a stronghold of jaguars in the Biome, as previously reported [24]. According to this, either a better management of the existing corridor or new bridges to the other PJCUs must be of priority for implementation in short-term. In this way, continuous assessment of wildlife can be helpful for evaluating the viability of such areas including the legal corridor. Based on our criteria PJCUs # 8 and 10 were classified as low priority for jaguar conservation. Yet we stress the need of accumulating information in this area since local people have reported jaguar presence.

The integration of spatially explicit models with expert opinions can assist in the identification and prioritization of sites such as core areas and potential corridors [71]. In this study, species distribution modeling technique were crucial for selecting core areas as to identify main environmental factors driving jaguar presence in the Caatinga biome. Expert opinions contribute with the construction of the permeability matrix and final designed corridors can be considered feasible. Besides carnivores have been used as focal species for connectivity modeling, we should be careful when modeling connectivity in a broad range, using the jaguar as focal species, since many factors can influence its presence and movement pattern across its distribution range. Previous study has designed jaguar corridors on a global scale using a slightly different approach [19]. Our study is zooming in a particular area of the distribution range of the jaguar and presents a comprehensive conservation plan for the species in the Caatinga biome, complementing and strengthening previous findings.

Although the creation of protected areas are more urgent and significant initiative to biodiversity conservation, this strategy will only be able to partially mitigate the problem. In this context, corridors can complement the role of protected areas, increasing the ecological function by means of bridging viable areas to biodiversity conservation. With the creation of corridors, government is able to regulate the land use within its areas favoring jaguar movements and resulting on the increase of the species population viability in the biome.

In conclusion, we emphasize the urgency of establishing a protected unit at the PJCU #1 and corridors with PJCUs # 4, 5 and 7, otherwise, we expect the most important jaguar population currently found in the biome to be extirpated and, consequently, disrupt predator-prey interactions affecting the entire ecosystem functioning [72].

## Supporting Information

Table S1 **Occurrence data of jaguars used to species distribution modeling, by site and/or city (Datum SAD69).**
(DOCX)

Table S2 Occurrence data of jaguars used to validation*, by site and/or city (Datum SAD69).
(DOCX)
