# Supplementary material for: Identification of Priority Conservation Areas and Potential Corridors for Jaguars in the Caatinga Biome, Brazil
Source: PLoS One. 2014 Apr 7;9(4):e92950. doi: 10.1371/journal.pone.0092950 (PMC3977835; doi:10.1371/journal.pone.0092950)
Supplement: Table S1 — Occurrence data of jaguars used to species distribution modeling, by site and/or city (Datum SAD69). (DOCX) [file pone.0092950.s001.docx]

Table S1. Occurrence data of jaguars used to species distribution modeling, by site and/or city (Datum SAD69).

| County/  State | ID | Site | Longitude | Latitude |
| --- | --- | --- | --- | --- |
| Anísio de Abreu - PI | 52 | - | -43.0584100 | -9.1903600 |
| Barra - BA | 15 | - | -43.1486400 | -11.0926900 |
| Bom Jesus da Lapa - BA | 5  6 | Paratinga  Boca do Riacho | -42.9461500  -43.3444700 | -13.1909200  -13.1892800 |
| Bonfim do Piauí - PI | 54 | - | -42.8759000 | -9.1716200 |
| Campo Formoso - BA | 24  26  27  34  39 | Laje dos Negros  Cambão  Mata Roçada  Careta  Baixa do Capim | -40.1666800  -41.1401860  -41.1100270  -41.0462180  -40.9634130 | -10.3500400  -10.2138110  -10.1841920  -9.9621900  -9.9071120 |
| Caracol – PI | 53  55 | -  - | -43.3745000  -43.4125000 | -9.1740000  -9.0804000 |
| Chorrochó - BA | 56 | - | -39.2000000 | -9.0515800 |
| Coronel José Dias - PI | 58 | - | -42.5036100 | -8.8726500 |
| Dom Inocêncio - PI | 57 | - | -41.9846000 | -9.0156300 |
| Gentio do Ouro - BA | 14 | - | -42.5473800 | -11.4782200 |
| Igaporã - BA | 2, 3, 4 | - | -42.6743403 | -13.8809926 |
| Itaetê - BA | 7  8 | -  Assentamento Roseli Nunes | -41.0411300  -41.1508000 | -13.0461200  -12.9678000 |
| Jeremoabo - BA | 28  42 | -  ARIE¹ Corocobó | -38.6674000  -38.8333000 | -10.0736100  -9.8333000 |
| João Costa – PI | 61 | - | -42.4485800 | -8.5129700 |
| Juazeiro - BA | 47 | - | -40.7164700 | -9.5788700 |
| Jurema – PI | 51 | - | -43.1263700 | -9.2305900 |
| Mirangaba – BA | 18  19 | - | -40.5703300  -41.2972800 | -10.954600  -10.722250 |
| Morro do Chapéu – BA | 11, 12, 13 | Sítio Adalberto  Cana Bravinha  - | -41.2479000  -41.2270000  -41.2116200 | -11.673800  -11.607800  -11.509870 |
| Ourolândia – BA | 17 | - | -41.1372900 | -10.981540 |
| Palmas do Monte Alto – BA | 1 | Palmas de Monte Alto | -43.1594100 | -14.269140 |
| Palmeiras – BA | 9 | Chapada Diamantina | -41.5000000 | -12.666700 |
| Pilão Arcado – BA | 31  49 | -  - | -42.4758600  -43.6143600 | -10.017720  -9.5036800 |
| Remanso – BA | 48 | - | -42.2666600 | -9.5167100 |
| São Raimundo Nonato – PI | 59  60 | Corredor Capivara-Confusões  Serra da Capivara | -42.8793800  -42.6310200 | -8.7031600  -8.6151700 |
| Seabra – BA | 10 | Marimbus-Iraquara | -41.7079600 | -12.3991300 |
| Senhor do Bonfim – BA | 23 | - | -40.2309300 | -10.3923900 |
| Sento Sé - BA | 20  22  25  29  30  32  33  35  36  37  38  40 | Serra Branca  Campo Largo  Minas do Mimoso  Lagoa do Mari  Limoeiro  Riacho Grande  Cumbri  Ciposão  São Romão  Brejinho  São Romão  Palmeira | -41.7503500  -41.4153800  -41.4181300  -41.6276600  -41.3403200  -41.0676380  -41.9342100  -41.0778780  -41.1566700  -41.5165700  -41.1748700  -41.6669000 | -10.6874900  -10.3926700  -10.2671700  -10.0655600  -10.0469100  -10.0104770  -10.0009000  -9.9614860  -9.9343800  -9.9255500  -9.9150000  -9.8971800 |
| Sobradinho - BA | 41  43  45 | Riacho Pontuó  São João  Bonsucesso | -40.9753000  -40.9749000  -40.9836400 | -9.8746500  -9.8309500  -9.7632700 |
| Tamboril do Piauí – PI | 62 | - | -42.8883700 | -8.3570100 |
| Uaua - BA | 44  46 | Jovelina  Jovelina | -39.4486400  -39.2756000 | -9.8129200  -9.7245000 |
| Umburanas - BA | 21 | Poço Burro | -41.8433600 | -10.5231700 |
| Várzea Branca - PI | 50 | - | -42.9700100 | -9.2342400 |
| Xique-Xique - BA | 16 | - | -42.9903700 | -11.0328100 |
