# Supplementary material for: Identification of Priority Conservation Areas and Potential Corridors for Jaguars in the Caatinga Biome, Brazil
Source: PLoS One. 2014 Apr 7;9(4):e92950. doi: 10.1371/journal.pone.0092950 (PMC3977835; doi:10.1371/journal.pone.0092950)
Supplement: Table S2 — Occurrence data of jaguars used to validation*, by site and/or city (Datum SAD69). (DOCX) [file pone.0092950.s002.docx]

Table S2. Occurrence data of jaguars used to validation*, by site and/or city (Datum SAD69).

| County /  State | ID | Site | Longitude | Latitude |
| --- | --- | --- | --- | --- |
| Campo Formoso – BA | 16 | Gameleira Duardo | -40.9355387 | -9.9448510 |
|  | 17 | Gameleira Duardo | -40.9127532 | -9.9386632 |
|  | 19 | Bebida | -41.0765048 | -10.1433677 |
| Morro do Chapéu – BA | 3 | Boa Esperança | -41.2262917 | -11.9204397 |
|  | 20 | Boa Vista | -40.9829193 | -11.5395407 |
|  | 22 | Cristal | -41.3131261 | -11.8251259 |
|  | 24 | Manoel Preto | -41.2717851 | -11.4070278 |
|  | 36 | Seu Nilton | -41.2596870 | -11.2698455 |
|  | 37 | Grota da Onça | -41.2741694 | -11.4177183 |
|  | 44 | Cachoeira do Ferro Doido | -40.9993456 | -11.6248240 |
|  | 49 | Ventura | -40.9947813 | -11.6738895 |
|  | 52 | Riacho Sítio do Padre | -41.2221732 | -11.4080764 |
|  | 55 | Serra Pé do Morro | -41.2012632 | -11.5760331 |
| Sento Sé - BA | 1 | Agua Azeda | -41.1956383 | -10.0941837 |
|  | 47 | Mundo Novo | -41.1305256 | -9.9365706 |
|  | 2 | Alegre | -41.4851917 | -10.3287648 |
|  | 7 | Careta | -41.0543949 | -9.9314513 |
|  | 8 | Careta | -41.0313244 | -9.9661488 |
|  | 9 | Careta | -41.0478513 | -9.9611125 |
|  | 10 | Careta | -41.0430776 | -9.9676228 |
|  | 12 | Careta | -41.0627609 | -9.9280102 |
|  | 13 | Ciposão | -41.0531136 | -9.9613243 |
|  | 32 | São Romão | -41.1586543 | -9.9427264 |
|  | 33 | São Romão | -41.1763631 | -9.9419880 |
|  | 34 | São Romão | -41.1892231 | -9.9222728 |
|  | 38 | Grota do Mateus | -41.4357416 | -9.9661902 |
|  | 39 | Lagoa do Mari | -41.5069062 | -10.1739685 |
|  | 40 | Limoeiro | -41.3269745 | -10.1679593 |
|  | 41 | Limoeiro | -41.3103817 | -10.1425820 |
|  | 42 | Limoeiro | -41.3170573 | -10.1563532 |
|  | 45 | Morro Solto | -41.0191628 | -9.9605279 |
|  | 46 | Morro Solto | -41.0295706 | -9.9626885 |
|  | 50 | Riacho Grande | -41.0419804 | -9.9817757 |
|  | 51 | Riacho Grande | -41.0382597 | -9.9992802 |
|  | 53 | Serra da Bananeira | -41.6491006 | -10.1523380 |
|  | 54 | Serra do Otaviano | -41.6028268 | -10.5862345 |
|  | 56 | Barra Baixio | -41.5380812 | -10.3886139 |
| Sobradinho – BA | 28 | São João | -40.9580241 | -9.8634813 |

*Only 34 independent-points were used in modeling.
